# Developing a placebo-controlled trial in surgery: Issues of design, acceptability and feasibility

**DOI:** 10.1186/1745-6215-12-50

**Published:** 2011-02-21

**Authors:** MK Campbell, VA Entwistle, BH Cuthbertson, ZC Skea, AG Sutherland, AM McDonald, JD Norrie, RV Carlson, S Bridgman, KORAL study group

**Affiliations:** 1 Health Services Research Unit, University of Aberdeen, Aberdeen, UK; 2 Social Dimensions of Health Institute, Universities of Dundee and St Andrews, UK; 3 Sunnybrook Health Sciences Centre, University of Toronto, Ontario, Canada; 4 Department of Surgery, University of Aberdeen, Aberdeen, UK; 5 Robertson Centre for Biostatistics, University of Glasgow, Glasgow, UK; 6 Centre for Medical Education, University of Edinburgh, Edinburgh, UK; 7 University Hospital of North Staffordshire NHS Trust, Staffordshire, UK

## Abstract

**Background:**

Surgical placebos are controversial. This in-depth study explored the design, acceptability, and feasibility issues relevant to designing a surgical placebo-controlled trial for the evaluation of the clinical and cost effectiveness of arthroscopic lavage for the management of people with osteoarthritis of the knee in the UK.

**Methods:**

Two surgeon focus groups at a UK national meeting for orthopaedic surgeons and one regional surgeon focus group (41 surgeons); plenary discussion at a UK national meeting for orthopaedic anaesthetists (130 anaesthetists); three focus groups with anaesthetists (one national, two regional; 58 anaesthetists); two focus groups with members of the patient organisation *Arthritis Care *(7 participants); telephone interviews with people on consultant waiting lists from two UK regional centres (15 participants); interviews with Chairs of UK ethics committees (6 individuals); postal surveys of members of the British Association of Surgeons of the Knee (382 surgeons) and members of the British Society of Orthopaedic Anaesthetists (398 anaesthetists); two centre pilot (49 patients assessed).

**Results:**

There was widespread acceptance that evaluation of arthroscopic lavage had to be conducted with a placebo control if scientific rigour was not to be compromised. The choice of placebo surgical procedure (three small incisions) proved easier than the method of anaesthesia (general anaesthesia). General anaesthesia, while an excellent mimic, was more intrusive and raised concerns among some stakeholders and caused extensive discussion with local decision-makers when seeking formal approval for the pilot.

Patients were willing to participate in a pilot with a placebo arm; although some patients when allocated to surgery became apprehensive about the possibility of receiving placebo, and withdrew. Placebo surgery was undertaken successfully.

**Conclusions:**

Our study illustrated the opposing and often strongly held opinions about surgical placebos, the ethical issues underpinning this controversy, and the challenges that exist even when ethics committee approval has been granted. It showed that a placebo-controlled trial could be conducted in principle, albeit with difficulty. It also highlighted that not only does a placebo-controlled trial in surgery have to be ethically and scientifically acceptable but that it also must be a feasible course of action. The place of placebo-controlled surgical trials more generally is likely to be limited and require specific circumstances to be met. Suggested criteria are presented.

**Trial registration number:**

The trial was assigned ISRCTN02328576 through http://controlled-trials.com/ in June 2006. The first patient was randomised to the pilot in July 2007.

## Background

The placebo-controlled trial is a widely accepted design for evaluating pharmacological and device interventions. There has, however, been considerable debate in the literature about the ethical acceptability of including a placebo in procedures such as surgery. Whilst the use of a placebo in surgical trials is not new [1-8] the concept remains highly controversial. Several commentators have argued that placebo procedures are ethical for certain trials of surgery [9], but others have argued strongly that the use of surgical placebos cannot be justified as any surgical procedure carries risks of harm that are greater than those associated with no surgery [10,11].

The term "placebo" is commonly used to describe any substance or procedure a patient accepts as medicine or therapy, but which has no known mechanism other than a patient's belief in its value [12]. The aim of any placebo is to maximise the mimic of the active intervention (and its benefits) whilst minimising the risks associated with it [13,14]. A range of interventions, from dummy pills to surgical techniques, have been used as placebos [14]. Within a surgical context, however, no surgical placebo can be completely without the possibility of harm. This leads to particularly complex issues when trying to design a surgical placebo-controlled trial.

In this paper we report on a study (the KORAL study) conducted to assess the design, acceptability, and feasibility issues relevant to designing a surgical placebo-controlled trial for the evaluation of the clinical and cost effectiveness of arthroscopic lavage (washing out of the knee space under general anaesthetic) for the management of people with osteoarthritis of the knee in the UK. Whilst the primary focus was on arthroscopic lavage (and was written up in a separate monograph [15]), the study highlighted a range of wider issues relevant to the design and conduct of surgical placebo-controlled trials in general, and it is those that we focus on in this paper.

### The KORAL study

Our group was commissioned by the UK National Institute for Health Research Health Technology Assessment (NIHR HTA) Programme to design and conduct a placebo-controlled trial to assess the clinical and cost effectiveness of arthroscopic lavage for the management of people with osteoarthritis of the knee in the UK. The purpose of the trial was to confirm or refute the findings of an earlier study conducted in the US by Moseley and colleagues [16]. In the Moseley trial, patients had been randomised to arthroscopic lavage, arthroscopic debridement or placebo procedure and had found that whilst all groups improved, no significant difference was observed at follow-up between the placebo group and either 'active' surgery group; the conclusion being that observed benefit was due to the placebo effect. Whilst the Moseley trial had been conducted with methodological rigour, it had been conducted in a single US centre by a single surgeon and the generalisability of the results had been questioned by a number of authors [17-19]. Given that it was unclear whether an acceptable placebo could be designed and delivered in a feasible manner, the project first explored in an in-depth manner issues around the design, acceptability and feasibility of a surgical placebo for this trial (it is this in-depth study that is presented in this paper).

This study, which was known as KORAL (Knee Osteoarthritis: Role of Arthroscopic Lavage) had the following research questions (RQs) that were addressed in a staged way in a series of sub-studies:

RQ1. Is there a need for a further placebo-controlled trial of arthroscopic lavage for osteoarthritis of the knee? If yes,

RQ2. Can an appropriate surgical and anaesthetic placebo be designed for such a trial? If yes,

RQ3. Would key stakeholders find the proposed placebo-controlled trial design acceptable? If yes,

RQ4. Would conducting such a multi-centre surgical placebo-controlled trial be feasible in the UK?

## Methods

The research was conducted in two main phases. Firstly, an in-depth qualitative and quantitative exploration of possible placebo designs and their acceptability to key stakeholder groups (addressing RQs1-3) was conducted. Secondly a formal pilot of the proposed trial design to test feasibility (RQ4) was undertaken. Multi-centre Research Ethics Committee (MREC) approval was received separately for the two phases. Full details of the methods used in this study were given in the clinical monograph [15], however, brief details are provided below.

### Exploration of possible placebo designs and the acceptability of a placebo-controlled trial to key stakeholder groups

In the first phase, we particularly addressed: a) the perceived scientific merit of further evaluation of arthroscopic lavage (including by placebo-controlled trial); b) the choice of the placebo procedure, both surgical and anaesthetic; and c) the likely acceptability of different placebo-controlled trial designs to key stakeholder groups including surgeons, anaesthetists, potential participants and chairs of ethics committees.

We conducted focus groups with, and postal surveys of, surgeons and anaesthetists; focus groups and interviews with people with osteoarthritis (potential trial participants); and interviews with Chairs of MRECs (Table 1). Focus group discussions were informed by a presentation from the project team on background rates of arthroscopic lavage, details of the Moseley trial (including the design, results and perceived criticisms) and the project brief. Focus group discussions and interviews were audio-tape recorded and transcribed. Transcripts were analysed thematically using a modified Framework approach [20]. Within the focus groups and interviews we used the term "placebo surgery" (rather than possible alternatives such as "sham" or "dummy" surgery) as early on in the research we found that the choice of word could lead to different perceptions, despite the rationale behind their use being the same [15]. The term "placebo surgery" was adopted in an attempt to describe as accurately as possible the intention behind the procedure ie, to maximise the mimic, whilst minimising the risk.

**Table 1:** Details of those who contributed to the focus groups, interviews and surveys

Study component	Number who participated
*Focus groups with health professionals:*	
• two surgeon focus groups at the 2005 British Orthopaedic Association meeting	16 surgeons
• one regional surgeon focus group	25 surgeons
• plenary discussion at the 2005 British Society of Orthopaedic Anaesthetists meeting	130 anaesthetists
• detailed focus group at the 2005 British Society of Orthopaedic Anaesthetists meeting	8 anaesthetists
• two regional focus groups with anaesthetists	50 anaesthetists
*Focus group and interviews with people with osteoarthritis:*	
• two focus groups with members of the patient organisation *Arthritis Care*	7 people
• telephone interviews with patients on consultant waiting lists from two UK regional centres	15 people
*Interviews with Chairs of UK MRECs:*	
• telephone interviews with MREC Chairs	6 MREC Chairs
*Surveys of health professionals:*	
• postal survey of all members of the British Association of Surgeons of the Knee	382 surgeons
• postal survey of all members of the British Society of Orthopaedic Anaesthetists	398 anaesthetists

Note: 12 of the 13 UK MRECs which were in existence at the time of the research were invited to take part in the research. (To preserve their independence with regard to any future ethics decisions about KORAL, the MREC that approved this part of the study was excluded from this component)

All members of the British Association of Surgeons of the Knee and members of the British Association of Orthopaedic Anaesthetists were surveyed for their opinion. Permission was received from both Societies for only a single mailing to members. Responses to the surveys were summarised using simple descriptive statistics.

The final output of this first phase was the template for a preferred trial design.

### Formal pilot of the proposed trial design to test feasibility

The second phase was a formal pilot of the preferred trial design that had been developed in Phase One. The formal pilot was conducted in two centres. Analysis of the pilot data consisted primarily of descriptive statistics including proportion of eligible patients randomised, and reasons for refusing to take part in the trial.

## Results

### Need for proposed further evaluation of arthroscopic lavage

From the focus groups and interviews, there was broad acceptance across all stakeholder groups of the need to find out more about the effects of arthroscopic lavage. Surgeons expressed uncertainty about the overall effectiveness of lavage. On the one hand some indicated that there was some evidence to suggest that lavage might offer at least short-term pain relief:

"if ... you end up washing the knee out, sometimes the symptoms do improve and make it pseudo-working. We had a lady recently, and she had a defect, and she's a lot better since we washed the knee out, that's three weeks ago now." (Surgeon 1, Group C)

However, others commented that they often observed their patients "all coming back" with continuing problems after the procedure, thus raising concerns about the longer term and overall effectiveness of the technique.

People with osteoarthritis of the knee also expressed the need to find out definitively whether arthroscopic lavage was effective. For example, one mentioned the scientific uncertainty surrounding the effectiveness of arthroscopic lavage, and another highlighted the need for research into the long-term effectiveness of such surgical procedures:

"Well, we have got to find out, you know, it has been going on for years and years and no one has ever found a complete answer so things have got to be tried, you know... If you want to advance that is what you have to do."(Participant 1)

"I certainly think it [a trial of arthroscopic lavage] is worthwhile because at the end of the day ...I don't think that people should undergo surgery unless it was having some long term benefit to them ... it should only be done when it is going to have a positive effect and a long lasting effect." (Participant 2)

Although all the groups accepted there was a need to find out more about the effects of arthroscopic lavage, there was variation in opinion about *how *researchers should investigate this and about whether it would be acceptable to investigate the effectiveness of arthroscopic lavage using placebo surgery. This is discussed below.

### Design of a surgical placebo

Discussion within the surgeons' focus groups concentrated mainly on the ways in which a placebo could mimic arthroscopic lavage (the active surgery), whilst ensuring that any risks of harm were minimised. The consensus emerged fairly readily that three superficial skin incisions were needed, that these should only pierce the epidermis, and that any penetration of the knee capsule should be avoided.

"No, you don't have to do the dermis ... just enough to make it bleed" (Surgeon 8, Group B)

Ensuring that penetration of the knee capsule did not occur was promoted for two primary reasons: a) that it would reduce the risk of any infection and b) it would ensure that no form of lavage was inadvertently performed:

"If you put the scope in you introduce fluid therefore technically it becomes a lavage even if it's a tiny amount, doesn't it?" [several yes's round the table] (Surgeon 7, Group C)

Within the anaesthetists' focus groups, the question about the most appropriate form of anaesthesia to incorporate within a placebo procedure was more contentious. Some anaesthetists objected to the ethics of conducting any research that involved a placebo (see acceptability section below), and did not feel comfortable discussing the design of an anaesthetic for such a procedure. However, a consensus eventually emerged that the patients in both trial groups should receive the same anaesthetic, and that this should be the regimen the individual anaesthetists who participated in the trial would customarily use for a simple arthroscopic procedure (i.e. a general anaesthetic). They believed that this would not only maximise the mimic of the active surgery but would also minimise the risks to participants. As they had more experience with general anaesthesia in these cases, they believed it would be safer than a technique using a combination of sedatives and analgesics as used in the Moseley trial (on the premise that it was less risky for their patients), but was felt to be less familiar and therefore less safe by our respondents:

"Hasn't the starting point got to be, you should do the same [as for the active surgery group] unless there is a really good reason not to? And if the really good reason is all about risk then you have to show that their [the sedation procedure used by the Moseley trial, on the grounds that it was of lower risk to patients] intervention has less risk than the standard full anaesthetic. I am convinced that that is not the case. So therefore you should do the standard straightforward general anaesthetic" (Anaesthetist 5, Group B)

"Statistically, [the sedation procedure used by the Moseley trial], is more dangerous than a general anaesthetic [on the grounds that it was of lower risk to patients]" (Anaesthetist 6, Group A)

Assuming that general anaesthesia was to be adopted, the anaesthetists within the focus groups agreed that inclusion should be restricted to low risk patients, as defined by those who were American Society of Anesthesiologists (ASA) grades 1 or 2 [21,22] - that is "normal healthy patients" or "patients with mild systemic disease" who had no other contraindication to anaesthesia.

### Acceptability of a surgical placebo-controlled trial

#### Views expressed by health professionals

Although none of the surgeons who took part in the focus groups disputed the need for further investigation of the effectiveness or otherwise of arthroscopic lavage, there was extensive debate within the groups about whether a placebo-controlled trial was necessary to generate new knowledge, and whether it was acceptable. For example:

"It would be more ethically correct to compare doing nothing to a lavage first and then look at the results and see"... You don't need to know the benefits of the placebo, it's irrelevant. When you make a clinical decision, you have to decide whether it's lavage or not. And so all you need to know is benefit from lavage and benefit from not doing anything and if the benefit from the lavage is marginal, then you don't do lavage and that's all that you need to do..."(Surgeon 3, Group C)

"What you need to do first is a decent study to actually look at conservative versus operative [management] and then once you've done that decent study, can you consider putting people at risk of placebo operations" (Surgeon 10, Group C)

Other surgeons disagreed, however, arguing that there was a methodological need for a placebo surgical trial because: a) a placebo component is needed to detect a small difference between the groups; and b) that a placebo is needed to attempt to disentangle what (if any) aspect of the arthroscopic lavage procedure is having a positive effect.

Overall, the health professionals tended to be split between: a) those who were strongly opposed to the inclusion of a placebo surgical arm on the grounds that it could lead to potential harm among individuals who could expect no personal benefit; and b) those who were in favour as that they believed the small risks that relatively few people in a placebo surgery trial arm would be exposed to were justified (because they were outweighed by the potential benefit to future patients and broader society of helping to ensure either that a demonstrably effective surgical procedure was used or that a demonstrably ineffective procedure was stopped).

Those opposed to the inclusion of a placebo surgical arm expressed strong personal views on their perceived ethics of such an approach:

"As an anaesthetist I would not anaesthetise someone for sham surgery. I just couldn't! I just think it's immoral and unethical ... I mean it's as simple as that, you wouldn't do it". (Anaesthetist 1, Group A)

"The number who will do this willingly will be very, very small, most of my colleagues would say - no you're joking"...(Anaesthetist 6, Group A)

On the other hand, those in favour pointed to the benefit to future patients and the desire to let patients rather than clinicians decide what was best for them:

"If the patient is prepared to accept the risk in order to have the operation and they are prepared to enter the trial on the understanding that they might not have an operation, are we all being a bit precious [ie, overly protective]?" (Anaesthetist 4, Group B)

"34,000 people ... per year are having a procedure which has no proof to it. So you're already doing the ladies with the [weak] hearts, putting the tourniquets up, giving them the drugs for absolutely no proven evidence... at the moment if there are 34,000 of these procedures being done and we are exposing that number of patients to all the risks of anaesthesia then we need to know the answer" (Anaesthetist 5, Group A)

Some individuals who were personally in favour of using a placebo were concerned that professional regulators would not be (with consequent implications for their potential participation):

"Interesting though ... I accept [it] is completely logical that the needs of the many outweigh the needs of the few but the GMC [General Medical Council] doesn't see that do they? The GMC make it very specific in their guidance to us that it is the needs of the individual which is your primary concern" (Anaesthetist 4, Group B)

One hundred and seventy three (43%) members of the British Association of Surgeons of the Knee responded to the survey as did 136 (34%) members of the British Society of Orthopaedic Anaesthetists (Table 2). Findings from the surveys supported the insights observed in the focus groups. The surveys showed that a sizeable percentage of health professionals (51% of surgeons and 40% of anaesthetists) were supportive of a trial with a placebo arm being mounted. The survey also showed that 43% of surgeons would personally consider taking part in such a trial as would 47% of anaesthetists. It was interesting to note that although some anaesthetists were personally not in favour of a placebo arm being involved they would, however, consider taking part if their surgeon colleagues wished to take part.

**Table 2:** Attitudes of surgeons and anaesthetists to a placebo controlled trial

	Surgeons	Anaesthetists
Number of questionnaires despatched	382	398
Number (%) of questionnaire returned	173 (45%)	136 (34%)
***Potential trial of arthroscopic lavage vs placebo surgery vs conservative management:***	**n/N (%)**	**n/N (%)**
• Supportive of trial with placebo arm being mounted	85/168 (50.6)	54/135 (40.0)
• Would consider taking part in a trial with a placebo arm	71/166 (42.8)	63/134 (47.0)
• Would encourage a friend or family member to sign up for a trial with a placebo arm	67/168 (39.9)	48/135 (35.6)

As part of the survey we also asked surgeons and anaesthetists for their views on the appropriate randomisation ratio for any potential trial. The majority favoured an allocation ratio of 1:1:1 to arthroscopic lavage, placebo surgery or non-operative management (60% of surgeons, 46% anaesthetists) or had no preference (25% surgeons, 41% anaesthetists), rather than a 2:1:1 ratio (10% surgeons, 10% anaesthetists) or some other ratio (5% surgeons, 3% anaesthetists).

#### Views expressed by people with osteoarthritis

In their focus groups and interviews, people with osteoarthritis echoed the need to find out more about the effects of arthroscopic lavage, and many of our sample indicated that they would consider taking part in a placebo-controlled trial. Two participants also discussed how, from a research point of view, including a placebo surgical component could be very useful. They drew on the information presented by the interviewers and explained that a placebo arm would help check whether any perceived benefit from arthroscopic lavage was due to a placebo effect. For example:

"... I would say it is important to have the placebo in it because if there is a sort of mind set that it does help to heal you, I mean it has been proven over the years that placebos do benefit in certain things." (Participant 1)

"... I think the placebo group is a very good idea because it can almost fool somebody into thinking they have had a procedure when they haven't and basically prove to some people that you think you are better because you think you have had this procedure but in fact you didn't have any treatment done at all."(Participant 2)

However, a few people in our sample thought involvement in a placebo-controlled trial would not be appropriate for them:

"if I was informed then that I had had the placebo and I realised that I had still got the pain I would be so furious...so angry" (Participant 14)

Those who were willing to take part openly acknowledged the risks of general anaesthetic and endorsed the need for anaesthetists to select only those at low risk. For example:

"... there is always, albeit I think it is quite small, risk of complications with anaesthesia ... there can be problems but they are very few and far between and if the right patients are selected then I don't think there would be any problems." (Participant 2)

#### Views expressed by Chairs of Ethics Committees

The Chairs of Ethics Committees highlighted a range of issues that should be addressed in any ethics application, eg, justifying the need for the placebo and the general anaesthetic, plans for minimising risks to patients, etc. Whilst they acknowledged that a surgical placebo-controlled trial would not simply be dismissed on principle, they predicted a "rough journey" through the ethics process for any such proposal:

"... I would have to think extremely laterally to envisage that this would get through without a very rough journey on the way ... We have one committee in particular which anything placebo ... is evaluated with a fine tooth comb and there we're talking little white tablets... The prospect of using a surgical approach I think raises the stakes enormously" (MREC 3)

"...*I would think that in conclusion it's probably the general anaesthesia that will cause ethics committees the most problems because then they will say now is this really too much of a risk to be giving somebody a general anaesthetic for nothing... I can see everybody say, 'Oh oh no way, not general anaesthetics'" (MREC 1)*

" ... I'd want a very robust justification for tackling the equipoise in this rather risky, in this potentially risky way. I think any self-respecting Committee that is the question they would ask. I would certainly be weighed by what risks our anaesthetic colleagues thought fair. I mean you've got to, you can't negate the risk... If the study is going ahead, there is a risk, you can't negate it. I think I'd want evidence that the risks had been fully considered and minimised... my experience is [that] anaesthetists are a very ethical lot indeed... and they serve as a very useful counterbalance to the surgeons ...I can see the surgeons are faced with people in awful, intractable pain and they want to do something about it" (MREC 4)

### Development of a preferred design to take to pilot study

**Figure 1:**
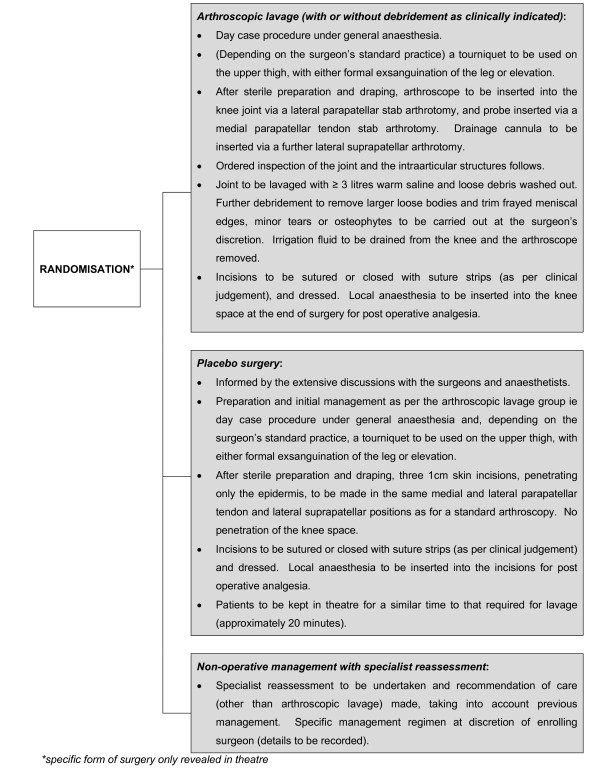
**Schematic of pilot**.

The final output of the exploratory phase was to develop a preferred trial design to take forward to a formal pilot. The preferred design was developed from the insights gained from the exploratory work undertaken with surgeons anaesthetists and potential participants. The finalised design was agreed with the funder and was as follows - patients were to be eligible for inclusion if they were: adults aged 18 years or older with radiological evidence of osteoarthritis of the knee who might be considered for arthroscopic lavage; at low risk for general anaesthesia - ASA grades 1 and 2; and able to give informed consent. Following consent, patients would be randomly allocated to: arthroscopic lavage (with or without debridement as deemed clinically necessary); placebo surgery; or non-operative management (fuller details of the three intervention arms are presented in Figure 1). No change in treatment (other than in analgesia use) was to be allowed in any of the randomised groups for a period equivalent to three months after randomisation.

For those randomised to some form of surgery, the type of surgery (whether arthroscopic lavage or placebo) was not to be revealed until the patient had been anaesthetised and was in the operating theatre. After surgery, discussions with patients would follow a pre-agreed approach: "*you know that I cannot tell you whether you had the active surgery or the placebo, but I can tell you that the procedure went well and we will now need to see how well this helps your knee*". This approach was to be maintained during follow-up clinic appointments.

### Feasibility of the proposed placebo-controlled design

#### Ethics approval

Gaining ethics approval for the pilot study was difficult and took nine months. The initial application for the pilot phase was rejected. Two main concerns were raised: a) the potential inclusion of surgeons who would not routinely offer arthroscopic lavage; and b) the potential inclusion of centres where arthroscopic lavage was being phased out and was no longer a routine treatment choice. We appealed against this decision on the counter-arguments that: a) surgeons who would *never *consider arthroscopic lavage would not agree to take part in the trial, so one could assume that all patients recruited from surgeons who agreed to participate in the trial would have a possibility (albeit sometimes low) of having been offered lavage had the trial not been in place; and b) it could have been uncertainty of effectiveness of arthroscopic lavage rather than certainty about the lack of effectiveness that led centres to stop undertaking *routine *arthroscopic lavage. Thus including those centres where surgeons who still wished to find out whether lavage was truly effective was a further justification for the research, rather than an ethical objection to it. The second MREC which heard our appeal approved the pilot, subject to our considerably extending the patient information leaflet (which we duly did).

#### Local authorisations

Despite ethics committee approval, the pilot subsequently required major discussion and negotiation at each individual centre before local clinical approvals could be obtained. Some of the arguments discussed at the ethics committee were raised again at local level and the fact that ethics approval had been granted did not mean that clinicians would automatically accept that the process was ethical. There were also concerns about who would pay for any placebo procedure and about indemnity arrangements. Despite extensive negotiations full local approval was not achieved for one of the two pilot centres within the four-month timeframe of the pilot study (a number, but not all, of the authorisations were successfully in place). In the centre where the pilot did receive approval, the local authorisation process also led to caveats being placed on the delivery of the trial locally (eg, the restriction that only consultant anaesthetists take part).

#### Delivery of the pilot design

**Figure 2:**
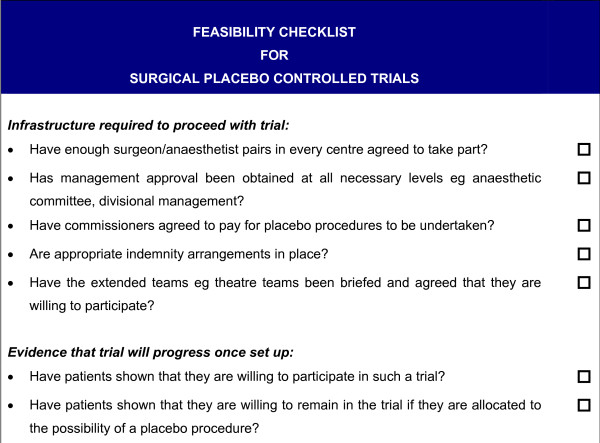
**Feasibility checklist for surgical placebo controlled trials**.

Eight clinics were held over the course of the pilot phase, drawing patients identified as potentially eligible for the trial by screening referral letters sent by the patients' General Practitioners (GPs). Forty nine patients were invited to attend. Of the 40 patients who attended, 13 were eligible and nine consented to take part (Figure 2). Six were randomised to some form of surgery and three to non-operative management. Two of the six patients randomised to surgery subsequently withdrew from the pilot prior to surgery. Both cited anxieties about the possibility of receiving placebo rather than active surgery among their reasons for withdrawal. One also highlighted concerns about Methicillin-resistant *Staphylococcus Aureus *(MRSA).

The three patients randomised to non-surgical management were reassessed by the treating clinician on the same day as the recruiting clinic, following randomisation. All three were advised on analgesic use. Two participants were given lifestyle modification advice and exercise information. The use of a walking stick was suggested to one participant but this was declined. Two participants were advised to use an elastic knee brace and one on the use of heat or ice.

One surgical session took place which involved one active and one placebo procedure (the two remaining surgical patients were managed outwith the framework of the pilot - see below). The patient allocated to active procedure underwent arthroscopic lavage with three litres of saline (debridement was not required) and the procedure was completed as per protocol. The placebo surgery was also undertaken successfully as per protocol. The surgeon and anaesthetist reported that the practicalities of both active and placebo surgery presented no major problems. Operating theatre staff did, however, express some concern when it was revealed that a patient was to receive placebo surgery (despite the fact that they had previously been fully informed of the nature of the trial). Through the two month follow-up questionnaire, neither patient reported that they thought they had undergone placebo surgery.

#### Decision whether to progress to a large-scale trial

Towards the planned end of the pilot, the funders reviewed our study findings to decide whether to continue seamlessly into the conduct of a full-scale trial. They concluded that a surgical placebo for arthroscopic lavage could be successfully designed, was generally acceptable to the range of stakeholder groups (although a few held strong views against the use of a surgical placebo under any circumstances), but faced considerable feasibility barriers when trying to conduct the trial in practice. In the light of these findings, the funders decided that the anticipated time, energy and cost required to bring multiple centres on board to recruit sufficient numbers (approximately 800 would be required) to a definitive large-scale trial over a sustained period of time was not justified (especially against a background of a gradual, albeit slow, decline in arthroscopic lavage [23]). As two of the pilot patients were still awaiting their surgery at the time of this decision, and it was as yet unclear if this was to be active or placebo surgery, it was deemed inappropriate to continue their management under pilot conditions and their management was reviewed outside the pilot framework.

## Discussion

Our study illustrated that a surgical placebo for arthroscopic lavage could be designed that was acceptable to a sizeable proportion of people across the range of stakeholder groups (although a few held strong views against the use of a surgical placebo under any circumstances), but conducting such a trial in practice would face considerable feasibility issues. Our study also illustrated well the opposing and often strongly held opinions that are held about placebos in surgery, the ethical issues that underpins this controversy, and the challenges of mounting such a trial that exist even when ethics committee approval has been granted.

### Issues of ethical and scientific acceptability of a placebo design in surgery

There was widespread acceptance in our study that further investigation was required into the effectiveness of arthroscopic lavage. Whether that investigation should or should not include a placebo-controlled design generated much wider discussion.

Commentators agree that the ethical principles appropriate to all clinical research must be satisfied as a minimum when considering a placebo-controlled design. These principles are that the study must: a) have scientific merit, b) be acceptable to participants in terms of the risk-to-benefit ratio of participation and c) respect the autonomy of participants by enabling them to determine whether they should participate [24]. In the qualitative components of our study participants raised and discussed these factors for the case of arthroscopic lavage. The scientific merit of the proposed study and the need for informed consent were readily accepted. Whether the proposed study design provided an acceptable balance of risks and potential benefits for participants generated much wider debate.

The discussion of an acceptable risk-to-benefit ratio in a placebo-controlled study is not straightforward. As Horng and Miller [25] argue, the risks must be considered in the context of alternative study designs to answer the research question - could an evaluation of arthroscopic lavage be conducted without the use of a placebo control and without compromising scientific rigour? The subjective measurement of the primary outcome in our study (patient reported pain) was recognised to be prone to bias in an open trial design (ie, when people would know which intervention they had been randomised to) [26], and it was considered impossible to maintain an open trial sufficiently long for any placebo effect to have dissipated, and as such the inclusion of a placebo control was deemed to maximise scientific rigour.

An additional consideration in the risk-to-benefit ratio for participants is the nature of the proposed placebo - how "risky" the proposed placebo is perceived to be. A placebo must be able to mimic the intervention under evaluation, but minimise the risks to those who might take part in the trial. Edwards *et al *suggest that perceptions of acceptability of placebo are likely to vary depending on the nature of the placebo in question [27]. In our study, agreement of the choice of placebo surgical procedure proved easier than the method of anaesthesia. The surgical approach chosen (three small skin incisions) was both a satisfactory mimic and had low intrusiveness and thus required little debate. However, the use of general anaesthesia, while an excellent mimic, was more intrusive and as such generated much greater discussion, and was the factor that caused most discussion with local decision-makers when seeking formal approval to conduct the pilot. It is interesting to note, however, that the content experts (ie, the anaesthetists) contended that the use of a general anaesthetic would be safer (because this is the technique which they used routinely and with which they have the greatest experience) than a supposedly less intrusive alternative, such as a form of analgeso-sedative regimen, with which they were less familiar. The wider literature supports this, suggesting that the success of a procedure is directly related to the number of procedures undertaken by that individual [28].

Further evidence of the controversial nature of the placebo design was the need to go to an appeal before the pilot trial received ethics committee approval, despite evidence of support from a range of surgeons, anaesthetists and potential participants. In response to the ethical debate raised by the Moseley trial [16], the American Medical Association produced a set of principles under which a placebo-controlled trial in surgery would be considered ethical [29]. These principles outlined: that surgical "placebo" controls should be used only when no other trial design will yield the requisite data; that particular attention must be paid to the informed consent process when enrolling participants in such trials; that the use of surgical "placebo" controls may be justified when an existing, accepted surgical procedure is being tested for efficacy (but that it was not justified when testing the effectiveness of an innovative surgical technique that represents only a minor modification of an existing, accepted surgical procedure); and that when a new surgical procedure is developed with the prospect of treating a condition for which no known surgical therapy exists, using surgical "placebo" controls may be justified, but must be evaluated in light of whether the current standard of care includes a non-surgical treatment and the benefits, risks and side-effects of that treatment. Our experience suggests that these principles remain relevant; but, for a placebo-controlled trial to be conducted successfully, it is clear that it must not only be an *ethically *and *scientifically *acceptable course of action but must also be a *feasible *course of action.

### Issues around feasibility of a placebo-controlled trial in surgery

Randomised controlled trials in surgery are well-known to be difficult to design and often suffer from recruitment problems [30,31] and adding a placebo component to the design adds to this complexity. Our study also faced a number of practical hurdles before it commenced recruitment and it proved impossible to surmount all of these in one of the two pilot centres within the four-month timeframe of the pilot. We found that: a) stakeholders in each trial centre needed to be fully briefed and any ethical and practical concerns resolved prior to trial commencement; b) that arrangements needed to be put in place to cover the costs of the placebo before the trial could go ahead; and that c) appropriate indemnity arrangements needed to have been instituted. The crucial importance of local stakeholders and gatekeepers has been outlined by a number of authors and the need to develop explicit recruitment and communication strategies identified [32,33].

Our study confirmed that recruitment to a surgical placebo-controlled trial is achievable - nine of 13 eligible patients approached agreed to join the trial - and the study also demonstrated that blinding of participants receiving surgery was successfully maintained. However, two of the six allocated to surgery subsequently withdrew before surgery citing concerns about the possibility of receiving placebo surgery and risks associated with hospitalisation as reasons. This raises questions about the potential influence of a placebo arm on retention rates in surgical trials.

This range of feasibility issues is likely to be faced by anyone considering a placebo-controlled surgical trial, and a checklist of appropriate issues that trialists should consider is presented in Figure 2.

### Strengths and weaknesses of the study

Surgical placebos are controversial and this is one of the few studies to have explored empirically the attitudes and perceptions of stakeholders on this important issue. In addition it sought to reflect the perspectives of a wide range of stakeholders including surgeons, anaesthetists, potential participants and ethics committee chairs. It also ensured UK-wide coverage of opinions through the professional surveys (although response rates were, like those for other surveys among health professionals, quite low), and this provides reassurance that the results are reflective of the current range of opinion on the issue of surgical placebos in general. Similarly the involvement of multiple centres in the research was a strength, as previous studies of placebos have often been conducted in a single centre setting. This was one of the key criticisms of the Moseley trial as it only involved a single surgeon in a single centre.

We recognise, however, that our study concentrated on a relatively minor surgical procedure and that the results may have been different if we had been trying to design a placebo for a more invasive procedure which would have required a larger surgical incision or more complex anaesthetic or a higher risk of major complications. We anticipate that in those circumstances consensus on both the design and acceptability of the placebo would have been harder to achieve and that recruitment to the study may have been lower. In addition, the number of patients recruited to the pilot study was small; limiting the conclusions we can draw from their responses. However, we are confident that the range of issues which require to be considered when planning a placebo-controlled trial in surgery were encountered in this study.

## Conclusions

Our study showed in principle, a placebo-controlled trial of arthroscopic lavage could be conducted in the UK, albeit with difficulty. It highlighted well that not only does a placebo-controlled trial in surgery have to be ethically and scientifically acceptable but that it also must be a feasible course of action.

In the light of our experience, the place of placebo-controlled surgical trials seems likely to be very limited. Our study suggests that the following conditions would need to be satisfied:

a) alternative designs would provide inferior (and potentially biased) results, particularly where the primary outcome is of a subjective nature and blinding cannot be sustained beyond the time of any placebo effect;

b) a placebo surgical procedure and type of anaesthesia can be devised which adequately mimic the active intervention with a level of intrusiveness and risk that is acceptable to the surgeons and anaesthetists who would take part in the trial, and to ethics committees, research governance assessors and potential participants;

c) appropriate practical arrangements can be instituted in local centres to ensure that the delivery of such a design would be feasible;

d) sufficient numbers of potential participants (after assessment of clear descriptions and careful explanations in patient information leaflets of the advantages and disadvantages of taking part) judge for *themselves *that the risk-to-benefit ratio of participation is acceptable to them; and

e) levels of compliance with the allocation are sufficiently high to sustain scientific rigour.

Those who would rule out the use of surgical placebos in these circumstances come up against two difficult questions: 1) What about the apparent acceptability of the methodology to potential participants? and 2) What do we tell people with this painful, chronic and progressive condition why a procedure that is unproven is being offered or why a potentially effective intervention has been discarded?

## Abbreviations

ASA: American Society of Anesthesiologists; GMC: General Medical Council; GP: General Practitioner; HTA: Health Technology Assessment; KORAL: Knee Osteoarthritis: Role of Arthroscopic Lavage; MREC: Multi-centre Research Ethics Committee; MRSA: Methicillin-resistant *Staphylococcus Aureus*; NCCHTA: National Coordinating Centre for Health Technology Assessment; NIHR: National Institute for Health Research; RQ: Research Question; UK: United Kingdom; US: United States.

## Competing interests

The authors declare that they have no competing interests.

## Authors' contributions

MC was the principal grant applicant, led on the development of the study protocol, led the writing of the manuscript, was responsible for the overall conduct of the study and is guarantor of the study. VE contributed to the development of the study design, led on the design of the qualitative component of the study, and contributed to the writing of the manuscript. BC contributed to the development of the study design, led on all anaesthetic and other clinical aspects of the study and contributed to the writing of the manuscript. ZS contributed to the development of the study design, was responsible for the day-to-day management of the qualitative component of the study, and contributed to the writing of the manuscript. AS and contributed to the development of the study design, led on all surgical aspects of the study and contributed to the writing of the manuscript. AMcD contributed to the design of the study materials, to the organisation of study authorisations and assisted in the preparation of the manuscript. JN contributed to the design and conduct of the study and assisted in the preparation of the manuscript. RC contributed to the design and conduct of the study, provided expert guidance on ethical arguments and the place of placebos within these frameworks and contributed to the writing of the manuscript. SB contributed to the design and conduct of the pilot at a local level and contributed to the preparation of the manuscript. The KORAL Study Group - Seonaidh Cotton, Angela Donaldson, Ray Fitzpatrick, Adrian Grant, Alastair Gray, James Hutchison, Marie Johnston, David Murray, Craig Ramsay, David Rowley, Luke Vale and Carlos Wigderowitz - were all members of the KORAL Project Management Group, advising on the design and conduct of the study, and commenting on drafts of the manuscript. All authors have seen and approved the final version of the manuscript.
